# Case report: A case of hyperthyroidism secondary to bone metastasis of differentiated thyroid cancer

**DOI:** 10.3389/fonc.2024.1354872

**Published:** 2024-02-26

**Authors:** Tingyu Gu, Zhihong Zhao, Yuanyuan Shi, Zhenhua Sun, Yao Wang, Zhiyuan He, Kun Wang

**Affiliations:** ^1^ Department of Breast and Thyroid Surgery, The Affiliated Hospital of Jiangsu University, Zhenjiang, Jiangsu, China; ^2^ Department of Pathology, The Affiliated Hospital of Jiangsu University, Zhenjiang, Jiangsu, China; ^3^ Department of Neurosurgery, The Second Affiliated Hospital of Soochow University, Suzhou, Jiangsu, China

**Keywords:** bone metastasis, secondary hyperthyroidism, differentiated thyroid cancer, diagnosis, pathology

## Abstract

It is usually believed that differentiated thyroid cancer is less likely to have distant metastases and rarely occurs secondary to hyperthyroidism. In our case report, we describe a patient diagnosed with thyroid fetal adenoma in 2002 who subsequently presented with a painful lump in her right rib. Through puncture biopsy, the mass was considered as metastatic follicular thyroid carcinoma, and then she appeared to have hyperthyroidism. The results of SPECT examination and other tests suggested that the hyperthyroidism was secondary to the thyroid cancer. The patient further underwent total thyroidectomy, and the pathology did not find any follicular thyroid foci. In this article, we analyze and discuss this case and review the relevant literature.

## 1 Introduction

Thyroid cancer (TC) is the most common malignant endocrine tumor in clinical practice, and recent data at home and abroad have shown that thyroid cancer is one of the most rapidly growing malignant tumors in terms of incidence (1). TC comprises several categories: ① differentiated TC (DTC), including papillary (PTC), follicular (FTC), and Huerthle cell tumors; ② medullary TC (MTC); and ③ anaplastic TC (2). DTC accounts for more than 90% of follicular cell-derived thyroid cancers (3). Approximately 85% of thyroid cancers are PTC, while FTC and Hurtle cell carcinoma together account for 5% of all thyroid cancers (4). A small number of DTC patients develop distant metastases, which are commonly found in bone, lung, brain, mediastinum, and liver and which seriously affect the quality of life and survival of patients. FTC is the most common histological type in patients with distant metastases (5). In this article, we report a case of differentiated thyroid cancer combined with multiple bone metastases as well as hyperthyroidism and analyze it to raise awareness.

## 2 Case data

**Figure 1:**
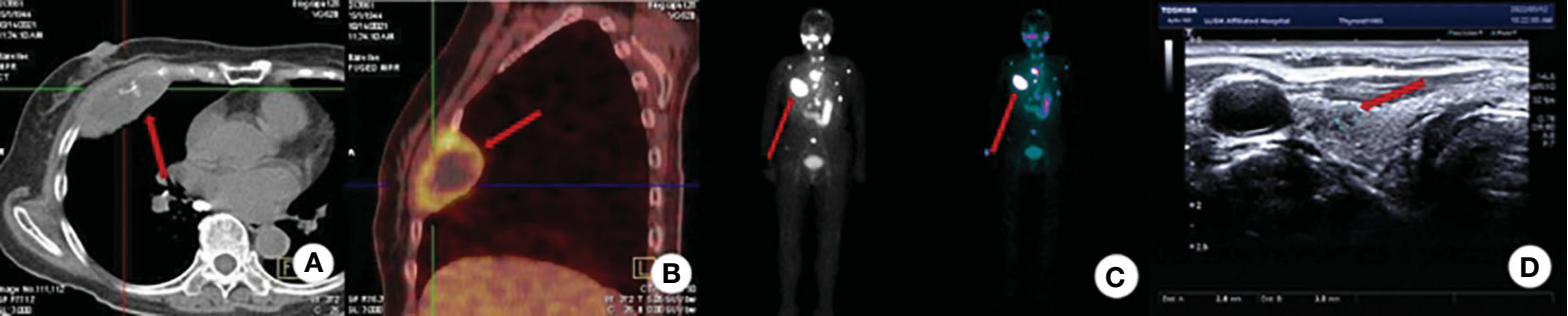
Imaging examination. **(A)** CT (axial view): Bone destruction of the 4th rib on the right side with a soft tissue mass shadow; **(B)** PET-CT: bone destruction of the right 4th anterior rib and T5 left arch with soft tissue mass shadow with increased metabolic activity, consider MT; **(C)** SPECT: Detection of multiple nucleoid-concentrated shadows in the field of view is considered for uptake of TC bone metastases. **(D)** Thyroid ultrasound: A nodule in the right lobe of the thyroid: C-TIRADS: Class 4A.

**Figure 2:**
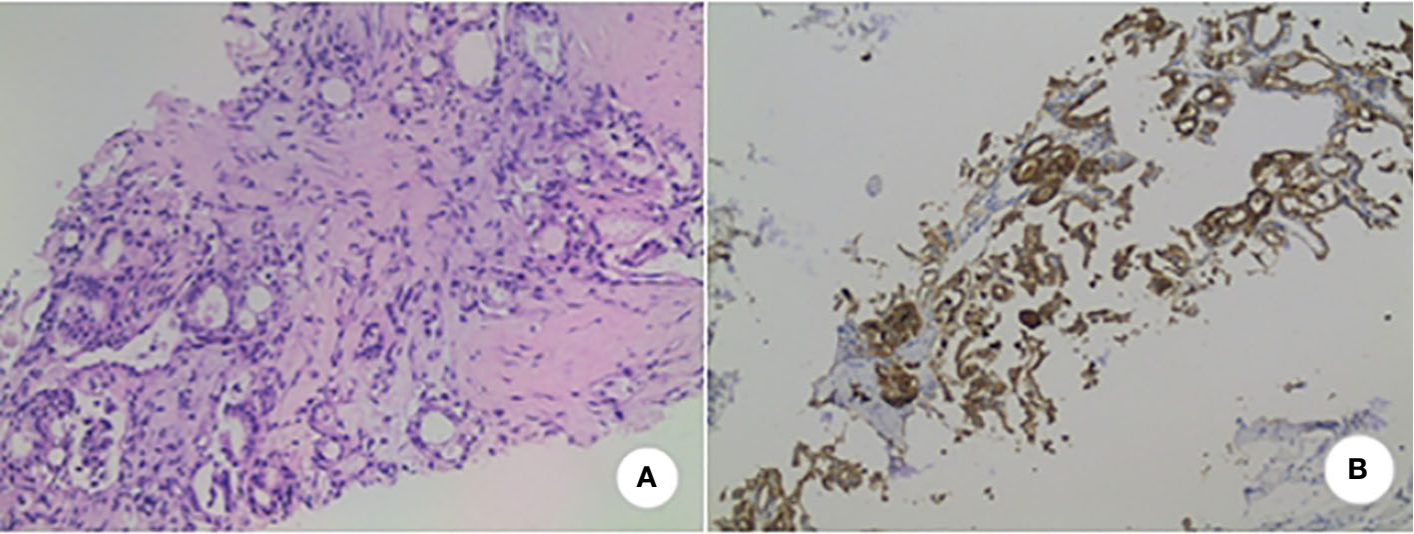
Pathology. **(A)** Follicular glands are seen in bone tissue as infiltrative growth, with crowded nuclei, some overlapping hairy glassy nuclei, and visible nucleoli. (HE stain, x200); **(B)** TG is positively expressed in tumor cells. (x200, Envision two-step method).

**Figure 3:**
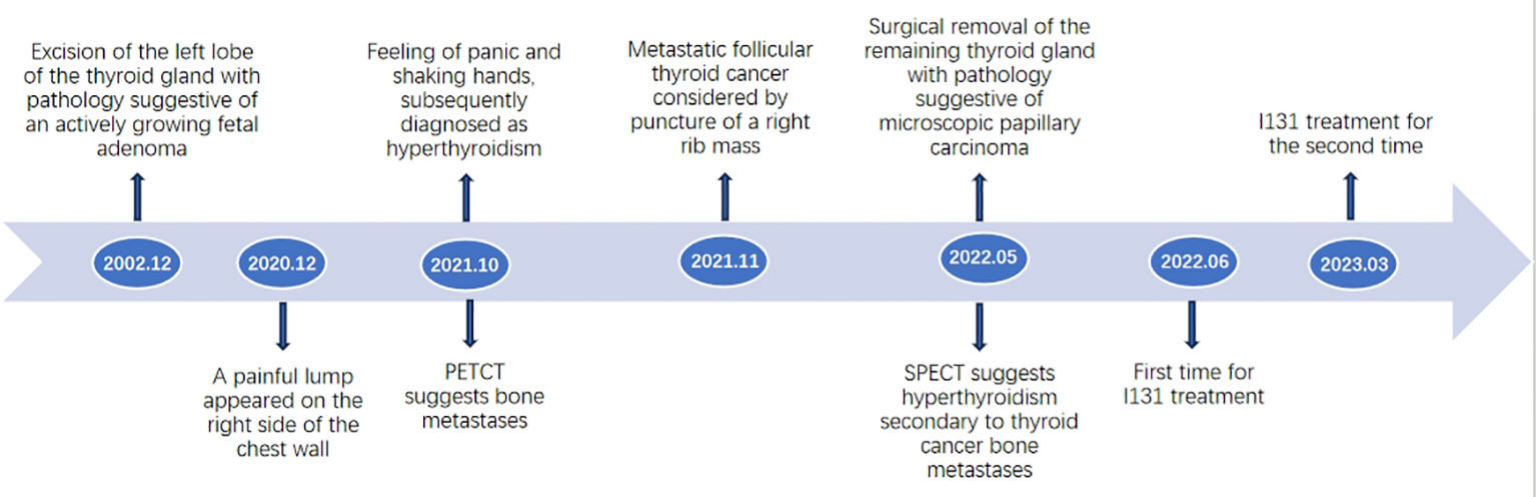
Timeline. A timeline of key points in the patient’s diagnosis and treatment.

The patient is a 78-year-old woman who underwent partial resection of the left lobe of the thyroid gland at an outside hospital in 2002 and was officially diagnosed with a fetal adenoma of the thyroid gland (with active growth) after the operation. In December 2020, the patient presented with a mass on the right side of the chest wall with pain. On October 11, 2021, the patient consulted the Department of Endocrinology of the hospital due to concern regarding warmth and tremor in both hands. By chemiluminescence, the thyroid hormone indicators were as follows: thyroid stimulating hormone (TSH), <0.005 µIU/ml (reference value 0.27-4.20μIU/mL); free thyroxine (FT4), 29.99pmol/L (reference value 12.00-22.00pmol/L); free triiodothyronine (FT3), 15.27pmol/L (reference value 3.10-6.80pmol/L); thyroglobulin (TG), >500.0 ng/ml (reference value 3.50-77.00 ng/ml). The endocrinologist diagnosed hyperthyroidism and proposed I131 treatment. While the iodine uptake rate showed that the 2-hour iodine uptake rate was 5%, the 6-hour iodine uptake rate was 5.9%, and the 24-hour iodine uptake rate was 6.3%. The patient’s iodine uptake rate was low, and she failed to undergo I131 treatment. PET-CT examination on October 14, 2021, revealed bone destruction of the right 4th anterior rib and the left arch of the T5 vertebra with soft tissue mass shadow, increased metabolic activity, suggesting MT, slightly increased bone density of the T5, T11, and L3 vertebral bodies, and slightly increased metabolic activity, suggesting metastasis (**Figures 1A, B**). In November, 2021, a further right rib biopsy was suggestive of thyroid tissue, and priority was given to metastasis of follicular thyroid carcinoma (**Figures 2A, B**). On May 6, 2022, the patient underwent SPECT examination that revealed a few residual glandular shadows in the left lobe of the thyroid gland; the right lobe had unevenly reduced nuclear continuity, and multiple clustered nucleoid-concentrated shadows were seen in the left parietal lobe of the thyroid gland, upper thoracic vertebrae, sternum, and anterior upper chest wall (**Figure 1C**). After endocrinological antihyperthyroidism treatment, the patient came to our department on May 11, 2023, for surgical treatment, and a hard mass of approximately 7*5 cm in size was palpable on the rib cage at the right fourth rib. Thyroid indicators (May 10, 2022) were as follows: TSH, <0.005μIU/mL; TG, >500.0 ng/ml; TgAb, 521.80 IU/ml; FT3, 11.76pmol/L. Thyroid ultrasound showed a hypoechoic nodule next to the larger nodule in the middle of the right lateral lobe of the thyroid gland, with a size of 2.8*3.0 mm, unclear margin, and irregular longitudinal shape (**Figure 1D**). On May 13, 2022, thyroidectomy of the right lobe, isthmus and left lobe residual thyroid was performed. Postoperative pathology suggested micropapillary carcinomas in the right lobe and isthmus and remnant benign thyroid tissue in the left lobe. The results of thyroid indicator testing (May 17, 2022) were as follows: thyroid hormones TSH, <0.005 µIU/ml; FT4, 55.18pmol/L; FT3, 19.68pmol/L. Currently, the patient has undergone two I131 treatments (June 2022 and March 2023), and the recovery has been fair. We created a timeline of key points in the patient’s diagnosis and treatment (**Figure 3**).

## 3 Discussion

DTC is one of the few head and neck cancers with a good prognosis, with a 10-year survival rate of up to 95% (6). A minority of DTCs develop bone metastases, and the incidence of bone metastases in patients with PTC has been reported to be approximately 6.9% (7); the size of the PTC nodule is closely associated with the occurrence of bone metastases, with the risk of bone metastases in a nodule larger than 4 cm being 54.1 times that in a nodule smaller than 1 cm (8). In contrast, the incidence of bone metastasis in FTC is approximately 28%, which is three times higher than that of PTC (9). In this patient, pain with a mass in the left chest wall was the first symptom, and PET/CT was performed, with results suggesting the possibility of bone metastasis of malignant tumors. Then, the rib biopsy suggested that bone metastasis of FTC was possible. Total thyroidectomy followed; no follicular carcinoma component was observed on postoperative pathology, and there were relatively small papillary carcinoma lesions. Therefore, combining the patient’s history of thyroid surgery in 2002 and postoperative pathology, we deduced that bone metastases were possibly due to metastasis of FTC.

Follicular carcinoma lacks the diagnostic features of papillary carcinoma. Well-differentiated follicular carcinomas are difficult to distinguish from adenomas, and multiple sections of the tumor and periphery are needed, with particular attention given to the presence of peripheral and vascular invasion. The new WHO classification in 2003 classified thyroid adenomas and their related tumors into follicular adenomas and vitelliform degenerative beam tumors, and fetal adenoma is the most common type of follicular adenoma in the world. Fetal adenoma is a poorly differentiated type of follicular adenoma that consists of tiny follicles with trabecular structures in an obviously edematous mesenchymal background and cuboidal epithelial cells that resemble fetal thyroid tissue (10). There was no clear delineation of fetal adenomas under the WHO classification prior to 2003, and the pathology report of this patient’s 2002 surgery suggested that the growth of fetal adenomas was active. Therefore, it was inferred that the pathology at that time was actually FTC. However, due to the problem of specimen preservation, it was not possible to obtain the time-relevant section or specimen of the case.

Another feature of this case was the release of large amounts of thyroid hormones from the bone metastases, which caused the patient’s hyperthyroid symptoms. The patient’s SPECT examination was suggestive of secondary hyperthyroidism caused by the release of thyroid hormones from the bone metastases. It is generally believed that TC and hyperthyroidism are rarely associated, that it is relatively common for hyperthyroidism to coexist with DTC and that secondary hyperthyroidism caused by metastatic thyroid cancer is rare. The main point in the treatment of hyperthyroidism due to metastatic thyroid cancer is to avoid the potentially fatal hyperthyroid crisis, and reports have shown that treatment can be accomplished by combining multiple, divided, and small doses of radioactive iodine and anti-hyperthyroid drugs (11). For this patient, drug treatment was carried out, but the effect was poor, and then surgical treatment was chosen with three main purposes: first, clarification of the nature of the nodule in the right lobe; second, to search for the primary foci of bone metastasis; and last, surgical resection of the thyroid tissue to facilitate subsequent treatment of the metastases with I131. For the management of bone metastases, combined with the patient’s condition, the patient has undergone puncture biopsy to clarify the nature of the metastases and prepared for I131 treatment. When evaluating whether to surgically resect the bone metastases, considering the patient’s age and the risk of surgical resection, it was decided to perform I131 treatment first after communicating with the patient and his family, and then surgical resection if the treatment effect was not good, fortunately the patient’s treatment effect was satisfactory after two I131 treatments at present. Surgical intervention is usually not the first choice for patients with bone metastases, but in the case of pathologic fracture, spinal instability, spinal injury, etc., surgical resection of bone metastases can alleviate pain and dysfunction of the patient, and allow the patient who would otherwise require prolonged bed rest to move around, thus prolonging the survival period and reducing the burden of care. Therefore, the decision of whether or not to surgically remove bone metastases should be made in light of the patient’s overall condition (12, 13). In clinical work, for patients with DTC combined with hyperthyroidism, doctors should conduct further examination to exclude the presence of distant metastases. The first symptom, bone pain, should be more carefully identified. According to the literature, patients with DTC bone metastases generally have a poorer prognosis and a lower survival rate than patients with bone metastases from other distant sites, with a 10-year overall survival (OS) rate ranging from 13% to 21% (14). The conventional treatment of BM includes radioiodine (RAI), surgical resection, external beam radiotherapy (EBRT), and chemotherapy. Kondraciuk J et al. (15) retrospectively analyzed the prognosis of 74 eligible patients with bone metastases from thyroid cancer in a hospital from January 2000 to November 2016, and found that the median survival time after the diagnosis of BM was 92 months, 1-year survival rate was 83%, 3-year survival rate was 59%, and the median survival time after the diagnosis of BM was 92 months. EBRT treatment can significantly improve survival (3-year survival probability: 79%). Patients with RAI affinity have better survival outcomes (3-year survival probability: 96%), and patients with earlier bone metastases tend to live longer. In this case, the patient underwent I131 treatment twice after surgery in June 2022 and March 2023; after treatment, the patient’s bone metastases were substantially reduced by imaging, serum thyroid hormones were reduced, and hyperthyroid symptoms were effectively controlled.

Reviewing all previous papers (searched on PubMed), we excluded some with incomplete patient information, and some with content unavailable for various reasons, and finally screened 11 cases of bone metastasis of thyroid malignancy secondary to hyperthyroidism (**Table 1**). Nine of these cases were differentiated thyroid carcinomas, of which four were PTC, four FTC, and one case in which both papillary and follicular carcinoma foci were found. In addition to this there was one case of ATC and one case of HCTC. The age of the patients: 60.0 ± 6.8 years, with the oldest patient being 71 years old and the youngest 47 years old. Almost all patients had obvious bone metastases (bone pain, pathological fractures, etc.) and hyperthyroidism (palpitations, hand tremors, fear of heat, etc.) at the time of diagnosis. Ten patients received radioactive iodine treatment, after which seven patients improved, one patient had disease progression, and two patients died within a short time. One patient refused to receive any treatment and follow-up, so the prognosis is unknown.

**Table 1:** Hyperthyroidism secondary to bone metastasis of thyroid cancer: review of literature.

Study	Gender, age (y)	Clinical presentation	Surgery	Histotype	Treatment	Concomitant distant metastases	Outcome
M E Girelli, 1990 (16)	F, 66	Pelvic pain	Thyroidectomy	PTC	I131 and L-thyroxine	Pelvic metastasis and hypothyroidism	Remission
K Ikejiri, 1997 (17)	F, 59	Pelvic bone metastases	Thyroidectomy	PTC and FTC	I131 and TAE	Multisite bone metastases and hypothyroidism	Progression
M Brauckhoff, 2001 (18)	M, 59	Symptoms of hyperthyroidism	Thyroidectomy and de-bulking resection of the metastasis	FTC	I131	sacrum bone metastasis and hyperthyroidism	Remission
Vijay Kumar, 2005 (19)	M, 65	Neck mass and back pain	Brief surgical exploration of the thyroid	ATC	Steroids and radiation therapy	Bone metastases and hyperthyroidism	Died
Dr Seidlin, 2007 (20)	F, 51	Heart palpitations and lower back pain	Thyroidectomy and the resection of spinal bone metastasis	FTC	I131	Spinal metastasis and hyperthyroidism	Remission
M Tardy, 2007 (21)	F,71	Heart palpitations and tremors	Thyroidectomy	PTC	I131	Bone metastasis and hyperthyroidism	Remission
Eijun Nishihara, 2010 (22)	F, 59	Gait disturbance, hip pain	Thyroidectomy	FTC	13mCi radioiodine therapy	Multisite bone, lung metastases and hypothyroidism	Remission
Abdelhamid Biyi, 2016 (23)	M, 62	Left arm pain	Thyroidectomy	PTC	I131 and L-thyroxine	left humerus and right pelvic bone metastases and hyperthyroidism	Remission
Mariko Aoyama, 2017 (24)	F, 57	Heart palpitations and tremors	Thyroidectomy	FTC	I131 and L-thyroxine	Lungs and skull metastases and hypothyroidism	Remission
Li-Li Zhang, 2020 (25)	M, 47	Neck masses, low back pain and lower limb paraplegia	N/A	PTC	N/A	Multisite bone metastases, muscular metastasis and hypothyroidism	Not know
Nikola Besic, 2021 (26)	M, 64	Dyspnea and left hip pain	Thyroidectomy and resection of the 8th right rib	HCTC	I131 and radiotherapy, doxorubicin chemotherapy	Lungs, mediastinum, bones metastases and hyperthyroidism	Died

PTC, papillary thyroid cancer; FTC, follicular thyroid cancer; ATC, anaplastic thyroid cancer; HCTC, Hürthle cell thyroid cancer; y, years; M, male; F, female; N/A,Not Applicable.

## 4 Conclusion

This case provided the following insights: first, the differential diagnosis of FTC and thyroid adenoma needs to be taken seriously in clinical work; second, for patients with DTC combined with hyperthyroidism, it is necessary to investigate whether distant metastasis is involved; third, for hyperthyroidism caused by metastatic foci, treatment needs to be considered comprehensively, with the specific treatment, such as surgery, medication, and I131, being selected according to maximum patient benefit.

## Data availability statement

The original contributions presented in the study are included in the article/supplementary material. Further inquiries can be directed to the corresponding author.

## Ethics statement

The studies involving humans were approved by Ethics Committee of Jiangsu University Hospital(KY2023K1105). The studies were conducted in accordance with the local legislation and institutional requirements. The human samples used in this study were acquired from primarily isolated as part of your previous study for which ethical approval was obtained. Written informed consent for participation was not required from the participants or the participants’ legal guardians/next of kin in accordance with the national legislation and institutional requirements. Written informed consent was obtained from the individual(s) for the publication of any potentially identifiable images or data included in this article.

## Author contributions

TG: Writing – original draft. KW: Writing – review & editing. ZZ: Writing – review & editing. YS: Writing – review & editing. ZS: Writing – review & editing. YW: Writing – review & editing. ZH: Writing – review & editing.
